# A Novel Strategy for Fabricating a Strong Nanoparticle Monolayer and Its Enhanced Mechanism

**DOI:** 10.3390/nano9101468

**Published:** 2019-10-16

**Authors:** Jun Zhou, Xiaoqing Cao, Linlin Li, Xingcheng Cui, Yu Fu

**Affiliations:** 1 Key Laboratory for Anisotropy and Texture of Materials, School of Materials Science and Engineering, Northeastern University, Shenyang 110819, China; caoxiaoqingfly@163.com; 2 College of Sciences, Northeastern University, Shenyang 110819, Chinafuyu@mail.neu.edu.cn (Y.F.)

**Keywords:** gold, nanoparticles, self-assembly, monolayer, cross-linking

## Abstract

This work presents the preparation of cross-linking Au nanoparticle (NP) monolayer membranes by the thiol exchange reaction and their enhanced mechanical properties. Dithiol molecules were used as a cross-linking mediator to connect the adjacent nanoparticles by replacing the original alkanethiol ligand in the monolayer. After cross-linking, the membrane integrity was maintained and no significant fracture was observed, which is crucial for the membrane serving as a nanodevice. TEM (Transmission Electron Microscopy), UV–Vis absorption spectrum, and GISAXS (grazing incidence small angle X-ray scattering) were performed to characterize the nanostructure before and after cross-linking. All results proved that the interparticle distance in the monolayer was controllably changed by using dithiols of different lengths as the cross-linking agent. Moreover, the modulus of the cross-linking monolayer was measured by atomic force microscopy (AFM) and the result showed that the membrane with a longer dithiol molecule had a larger modulus, which might derive from the unbroken and intact structure of the cross-linking monolayer due to the selected appropriately lengthed dithiol. This study provides a new way of producing a nanoparticle monolayer membrane with enhanced mechanical properties.

## 1. Introduction

Freestanding nanoparticle (NP) ultra-thin membranes as a novel nanomaterial have been applied in various fields including integrated microcircuits, chemical sensors, and so on [1,2,3,4,5,6,7,8,9]. The multilayered structure of the membrane causes difficulties in studying its fundamental properties. Thus, research interest has led to a monolayer membrane due to its simple two-dimension structure and abundant physicochemical properties that can be easily regulated by the interaction between adjacent nanoparticles [10,11]. The NP monolayer membrane has been regarded as an appropriate object for fundamental research and has a good prospective in various applications.

The mechanical properties are crucial for the monolayer membrane to function in the long-term, especially when serving as a basic unit to fabricate integrated components or nano-devices [12,13,14]. Generally, the NPs monolayer membranes fabricated by traditional methods have a low ordering degree and weak interaction among nanoparticles. Therefore, such structured membranes are fragile and difficult to employ [15]. In addition, the low-ordering pattern in the membrane and the poor mechanical properties limit the area of fabricated membrane that is required for many special applications. So far, numerous methods have been developed to improve the mechanical properties of a NP monolayer membrane based on physical and chemical technology [16,17,18,19]. For instance, slightly heating the Au NPs self-assembled at the water–alcohol interface produced a firm and large (several square centimeters) freestanding monolayer [16]. The acquired monolayer membrane showed an obvious promotion in mechanical strength and presented a high structure stability. However, the ordering degree of the monolayer was sacrificed after heat treatment because of the fusion of some particles, leading to a change in geometric configuration and deteriorated physiochemical properties such as thermal conductivity. The strength of the monolayer can also be enhanced by applying chemistry methods such as using cross-linking to enhance the interaction between nanoparticles [17,18,19]. A typical cross-linking agent is the dithiol molecule containing the -SH group. Taking advantage of the strong Au–S bonding, the dithiol links adjacent nanoparticles together tightly and subsequently strengthens the nanomembrane [20]. Kowalczyk et al. utilized an alkane dithiol molecule chemically crosslinked NPs monolayer membrane that showed high stability against the corrosion of organic solvents. As a result, the membrane could be deposited onto various topography surfaces while maintaining its own structure [21]. Though effective to improve the strength, the cross-linking method starts from the monolayer of disordered nanoparticles. The random distribution of Au nanoparticles in the monolayer membrane deteriorates the electrical conductance and optical property, limiting its applications. In order to solve this problem, a monolayer of ordered NPs was introduced into the cross-linking reaction [22,23]. The dithiol molecules replaced the capping molecules on the surface of the nanoparticles by the exchange reaction and successfully linked adjacent nanoparticles [22]. However, most exchange reactions are performed in a harsh solution for ordered structure. In this scenario, offering a relatively rigid monolayer with ordered structure is necessary. We prepared a stable ordered NP monolayer by vacuum thermal treating a self-assembled Au NP monolayer [11,24,25]. In this process, the alkyl chains of the ligand molecule generate a deep interdigitation with each other, leading to a strong interaction between the nanoparticles and a good resistance against various solvents.

Based on this, we present the preparation of a Au monolayer using a thermally treated Au NP ordered monolayer. To do this, various dithiol molecules with different alkyl chain lengths were applied to replace the original ligand molecules by thee exchange reaction. TEM (Transmission Electron Microscopy) and GISAXS (grazing incidence small angle X-ray scattering) characterization demonstrated that the alkyl chain length of the dithiol molecule could effectively tune the interparticle spacing in the monolayer. Quantitative measurement of the cross-linking membrane by AFM (Atomic Force Microscope) showed that their Young’s modulus gradually increased from the original 204.5 MPa to 268.5 MPa, signifying the role that chain length plays in determining the mechanical strength of the monolayer. To our best knowledge, this is the first report on the strengthening mechanism of crosslinked membranes.

## 2. Materials and Methods

### 2.1. Materials and Instruments

Chloroauric acid (HAuCl_4_·4H_2_O), sodium borohydride (NaBH_4_), toluene (C_7_H_8_), acetone (C_3_H_6_O), n-hexane (C_6_H_14_), and alcohol (C_2_H_6_O) were obtained from Sinopharm Chemical Reagent Co. Ltd. (Shenyang, Liaoning, China) and were used without further purification. 1H, 1H, 2H, 2H-perfluorodecyltriethoxysilane (C_16_H_19_F_17_O_3_Si), 1-dodecanethiol (C_12_H_26_S), 1,2-ethanedithiol (C_2_H_6_S_2_), 1,3-dimercaptopropane (C_3_H_8_S_2_), and 1,6-hexanedithiol (C_6_H_14_S_2_) were purchased from Alfa Aesar (Ward Hill, MA, USA). 1,4-Butanedithiol (C_4_H_10_S_2_) and 1,5-pentanedithiol (C_5_H_12_S_2_) were acquired from Tokyo Kasei Kogyo Co., Ltd. (Tokyo, Japan) and were used as received. Teflon tape was purchased from SPi Supplies (USA). Grazing incidence small angle x-ray scattering (GISAXS, Shanghai, China) was carried on the Shanghai Synchrotron Radiation Facility (SSRF, Shanghai, China). Atomic force microscopy (AFM, Karlsruhe, Germany) was measured on a Nanosurf Easyscan 2. Transmission electron microscope (TEM) observations were performed on a JEOL JEM-2100F (Tokyo, Japan).

### 2.2. Methods

#### 2.2.1. Preparation and Phase Transfer of Au NPs

The Au NPs were prepared following the literature based on NaBH_4_ as a reduction agent [26]. First, 9.475 mL deionized water together with 95 μL HAuCl4/HCl mixture (50 mmol·L^−1^) were added into a 50 mL colorimetric tube under the vibration of a vortex mixer. Then, 425 μL NaOH/NaBH4 solution (50 mmol·L^−1^) was injected into the HAuCl_4_ mixture immediately, followed by immersing the colorimetric tube into boiling water for 2.5 min, before finally the Au NPs solution was obtained. To transfer the Au NPs from water to hexane, 1-dodecanethiol mixed with n-hexane was used to exact the Au NPs by shaking the solution for about 30 s. After that, the hexane phase turned dark red from colorless, which indicated that the Au NPs had transferred to the hexane successfully.

#### 2.2.2. Fabrication of Ultrathin Monolayer Au NP Membranes on Quartz Plate and Heat Treatment

Before the experiment, quartz plates (2 × 2 cm) were immersed into an ethanol solution and cleaned with absorbent cotton repeatedly, followed by soaking for 30 min in n-hexane. After ultrasonic cleaning for 10 min, the quartz plates were treated with plasma bombardment to remove organic pollutants and were then placed vertically into a regent bottle with 4 μL perfluorinated regent (1H, 1H, 2H, 2H-perfluorodecyltriethoxysilane). Next, the bottles with quartz substrates were heated for 5 h at 60 °C. The perfluorinated regent would volatilize and absorb on the surface of the substrates to form a perfluorinated monolayer, meaning the transformation of the surface of the quartz plates to go from hydrophilic to hydrophobic.

The fabrication of the Au NP monolayer membranes was as follows. Briefly, 150 μL of toluene was first dropped on the pretreated quartz plates and the Au NPs solution in hexane was dropped to the toluene gradually until the drop surfaces were completely covered with NPs. After the solution volatilized completely, a close-packed Au NP monolayer membrane was obtained. In this experiment, part of the Au NP arrays was heated for another 5 h at 80 °C under 200 Pa.

#### 2.2.3. Fabrication of Ultrathin Monolayer Au NP Membranes on Teflon Tape and Heat Treatment

Prior to use, the tape surface was treated by toluene wiping and dried in a vacuum oven for 30 min at 80 °C, which makes it able to retain a clean surface.

This assembly process was similar to the one described above. A close-packed Au NP monolayer was prepared on the tape surface. Additionally, one half of the Au NP membranes was put into centrifuge tubes, followed by heat treatment in a vacuum oven at 80 °C for 0.5 h under 200 Pa.

#### 2.2.4. Cross-Linking of Ultrathin Monolayer Au NP Membranes

The ligand exchange experiments were performed in an alcohol solution. Typically, a Au NP array prepared on two different substrates was immersed into a solution with 20 mmol·L^−1^ of the cross-linking agent away from light about 12 h (different cross-linking agents are listed in Table 1). After that, the Au NPs array was washed carefully with alcohol and dried in air. The obtained array was measured by UV–Vis (range = 400 ~ 900 nm, speed = 0.5 nm, maximum absorbance = 2.000 Abs.) and TEM. GISAXS (λ = 1.24 nm, incident angel = 0.2°, reflex angle = 0.4°) was also applied to obtain information about the change in interparticle distance.

## 3. Results and Discussion

### 3.1. Preparation of Au NP Membranes and Heat Treatment

**Scheme 1:**
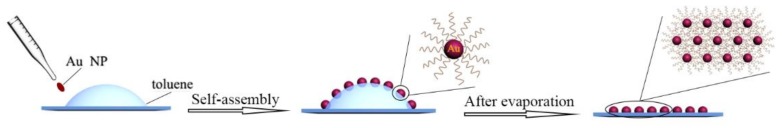
Schematic of the self-assembly process of the Au nanoparticles.

**Figure 1:**
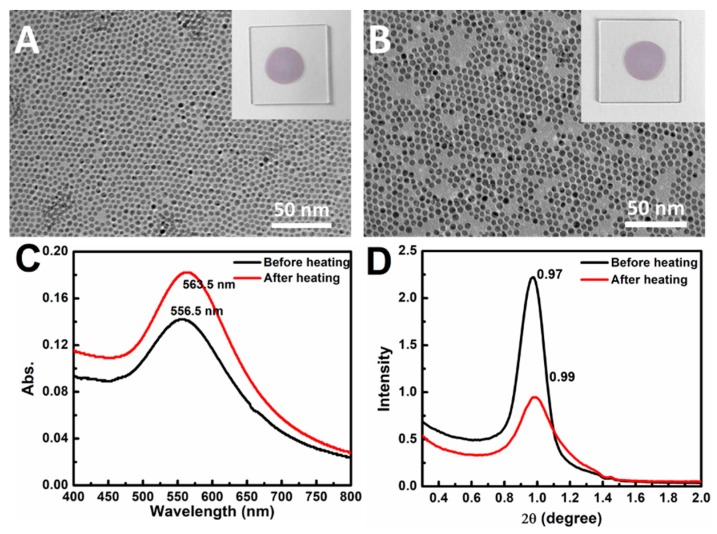
TEM images of the Au NP array fabricated on the quartz substrate (**A**) before and (**B**) after heating (inset is the optical photograph of array); (**C**) UV–Vis absorption spectrum; (**D**) SAXS (small angle X-ray scattering) curves of the Au NP array fabricated on the quartz substrate.

The Au NP membranes were prepared in accordance with the reported methods [26]. The detailed fabrication process is illustrated in Scheme 1. Prior to the experiment, a perfluorinated reagent was used to modify the quartz substrates in order to obtain a hydrophobic surface by the silane coupling reaction. After that, a drop of toluene was first dropped onto the substrate carefully and then Au NPs were spread on toluene to self-assemble at the toluene–air interface. After the toluene solution evaporated completely, a well-ordered Au NP monolayer array was deposited on the quartz substrate. TEM characterization results showed that the nanoparticles were close-packed to each other and the array presented a light pink without any visible wrinkle and breakage, as shown in Figure 1A. This phenomenon demonstrates that the prepared Au NP array possessed a regular internal structure and uniform external morphology. This assembly method is simple and highly reproducible, once it has satisfied the assembly conditions, which is the charged particles and an appropriate ligand molecule on the surface of the nanoparticles. The prepared gold nanoparticles are easily self-assembly at the toluene–air interface due to the equilibrium interaction deriving from electrostatic force and van der Waals forces. We believe that this method could be extended to other nanoparticles only if meeting the self-assembly condition on the toluene–air interface.

In order to enhance the monolayer stability, the prepared Au NP array was treated by vacuum heating at 80 °C for 0.5 h under 200 Pa. As shown in Figure 1B, the color of the Au NP array changed from red to purplish red and the packing of the particles became closer. Correspondingly, the UV–Vis measurement shows the maximum absorption wavelength (λ_max_) of the Au NP array’s red shift from 556.5 nm to 563.5 nm (Figure 1C), suggesting the enhanced surface plasma resonance (SPR) effect. This phenomenon might be derived from the decreased interparticle spacing, which is consistent with the results from the TEM characterization. Additionally, the sample was measured by SAXS, as shown in Figure 1D. It is obvious that the grazing angle increased from 0.97 to 0.99 along with the broaden peak shape after the heat treatment, indicating a decreased lattice constant and reduced structure order in the array. According to the reported literature [11], the NP interparticle distance (D) can be calculated by the following formulas (Equations (1)–(4)):

D = 2π/q,(1)

q = (q_x_^2^ + q_z_^2^)^1/2^, (2)

q_x_ = 4sin(θ_x_)/λ, (3)

q_z_ = 4sin(θ_z_)/λ, (4)

where D is the interparticle distance; q represents the diffraction vector; θ_x_ is from the peak value; and θ_z_ (0.2°) is obtained from the reflex angle. During the calculated process, q_x_ was replaced by the q. Here, the interparticle distance changed from 2.66 nm to 2.55 nm after heat treatment. All of the above results demonstrate that the interparticle distance is decreased by heat treatment. According to our previous report [24], the reduced distance between adjacent nanoparticles facilitated the interdigitation of the alkyl chain ligand and led to an improvement in the stability of the Au NP array.

### 3.2. Cross-Linking of Au NP Membranes

**Figure 2:**
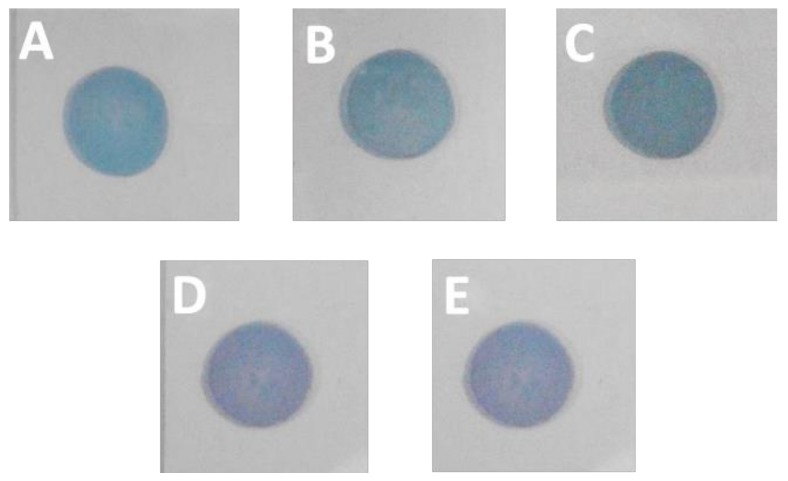
Photo images of the Au NP array cross-linked with ligands: (**A**) 1,2-ethanedithiol; (**B**) 1,3-dimercaptopropane; (**C**) 1,4-butanedithiol; (**D**) 1,5-pentanedithiol; (**E**) 1,6-hexanedithiol on the quartz substrate.

**Figure 3:**
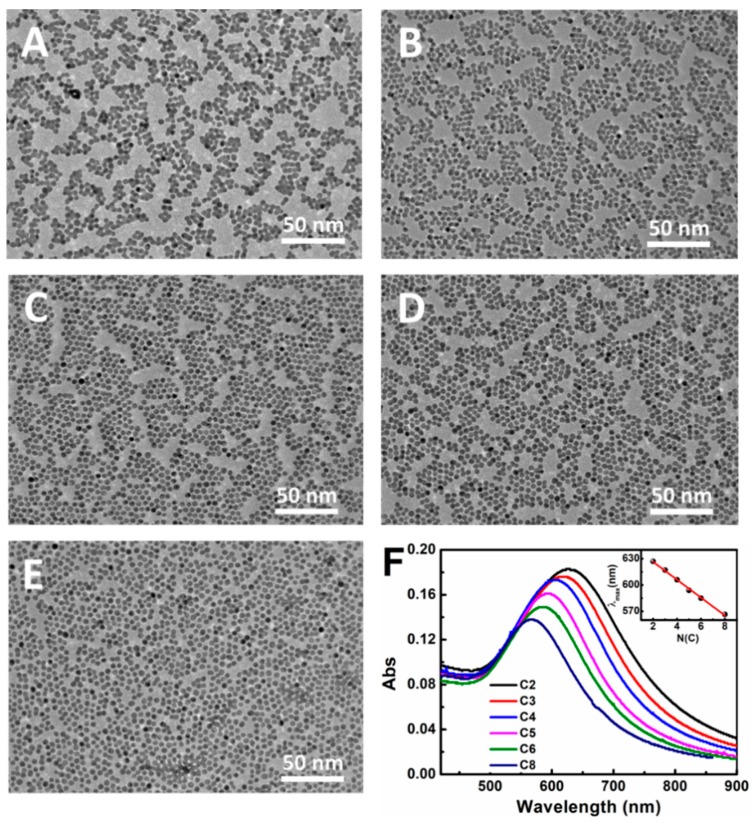
TEM images of the cross-linking monolayer prepared by the exchange reaction with different dithiol molecules: (**A**) 1,2-ethanedithiol; (**B**) 1,3-dimercaptopropane; (**C**) 1,4-butanedithiol; (**D**) 1,5-pentanedithiol; (**E**) 1,6-hexanedithiol; (**F**) UV–Vis absorption spectrum of the cross-linking monolayer with different dithiol molecules, inset is the plot of λ_max_ *vs* dithiol carbon numbers.

In order to obtain the cross-linking nanoparticles array, the preheated Au NPs array was immersed into an alcohol solution of dodecanethiolate (including 1,2-ethanedithiol (C_2_), 1,3-dimercaptopropane (C_3_), 1,4-butanedithiol (C_4_), 1,5-pentanedithiol (C_5_), and 1,6-hexanedithiol (C_6_)) to trigger an exchange reaction. In this process, the 1-dodecanethiol ligand was replaced gradually by the dithiol molecule due to the concentration effect in solution. As shown in Figure 2, the cross-linking arrays almost maintained their integrity without any obvious destruction in the macroscopic view. Compared with the preheated array, the cross-linking array presented a different color that changed from bluish violet to green, along with the decreased dodecanethiolate length. We concluded that the neighboring nanoparticles were dragged to approach each other by the short dithiol molecule, which resulted in a reduced interparticle spacing and enhanced LSPR (Localized Surface Plasmon Resonance) (representing the color change). This conclusion was also proven by the UV–Vis absorption spectrum measurement, as shown in Figure 3F. The λ_max_ of the Au NP array red shifted gradually with the decreased carbon numbers (C_2_ to C_8_). Detailed information about the λ_max_ *vs* carbon numbers is shown in the inset of Figure 3F. It is obvious that λ_max_ changed from 567 nm to 628 nm with the ligand changing from 1,8-hexanedithiol (C_8_) to 1,2-ethanedithiol (C_2_). These results demonstrate that the ligand exchange had been achieved and 1-dodecanethiol was successfully replaced by the dithiol molecule, which had a favorable regulation effect for the interparticle distance. Notably, the λ_max_ of the Au NP array and the carbon numbers (C_2_–C_8_) of the dithiol molecule presents a linear correlation. According to the literature, the length for the 1,6-hexanedithiol molecules was about 1.5 nm, which was nearly equal to the interparticle distance in the pre-heated Au NP array [10,26]. For the 1,8-octanedithiol, the long chain will not affect the array structure because of their flexibility property. Therefore, the exchange between the dithiol and 1-dodecanethiol hardly changed the interparticle distance, and hence would well maintain the structure of the Au NP array and LSPR property.

To further investigate the cross-linking array, TEM characterization was performed. As shown in Figure 3A–E, the array still remained as a single layer structure after cross-linking. Compared with the origin of the Au NP array, the interparticle distance of the crosslinked Au NP array decreased gradually, even appearing as a dumbbell structure (C_2_) with the reduced alkyl chain length of dithiol molecule. Meanwhile, the defect in the array begins to form and even generates a big cavity when a shorter dithiol is employed. All these results were consistent with the UV–Vis measurement, demonstrating the cross-linking reactions and their regulation effect for the interparticle spacing and array microstructure.

**Figure 4:**
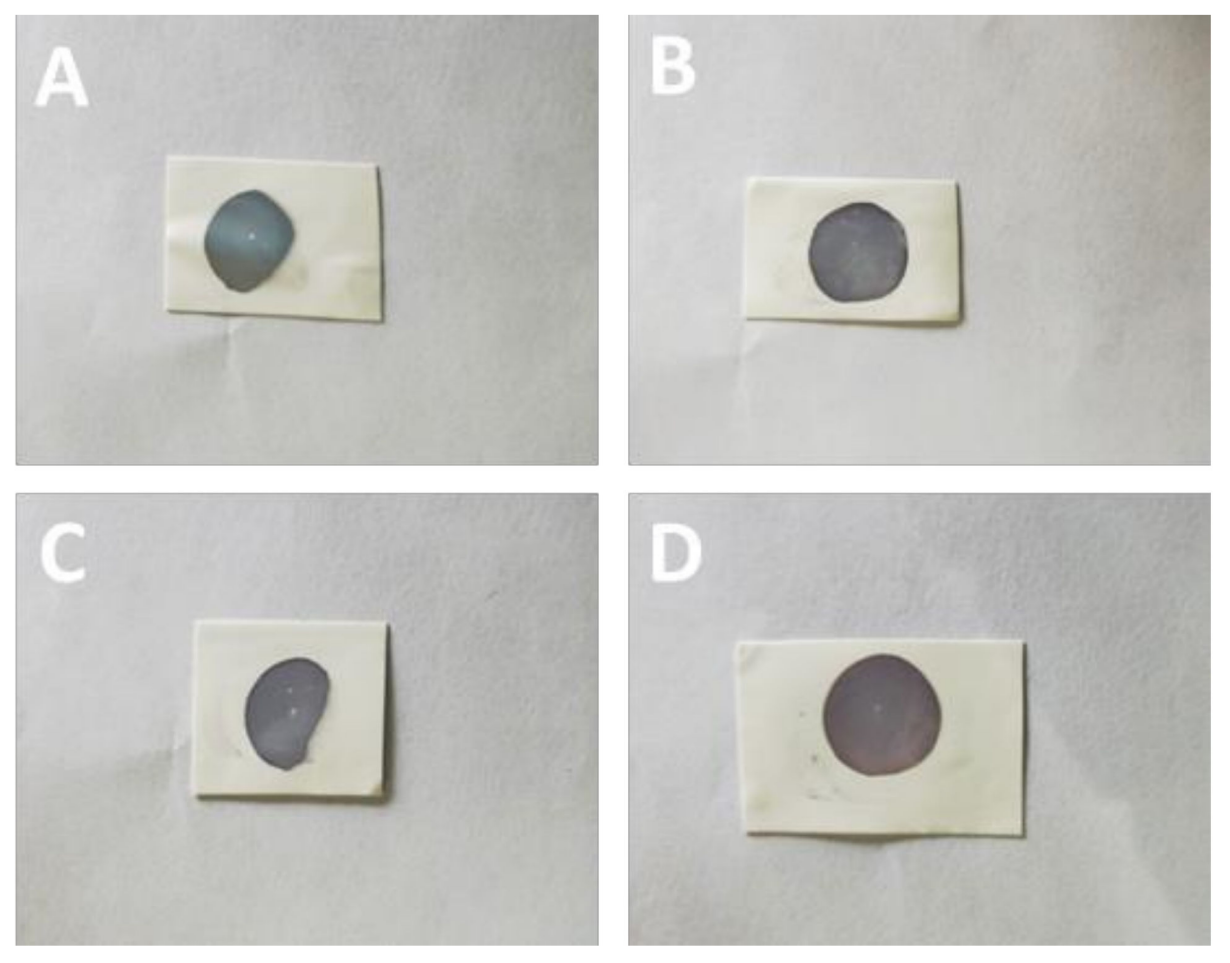
Photos of the Au NP array cross-linked with ligands on the Teflon substrate: (**A**) 1,2-ethanedithiol; (**B**) 1,4-butanedithiol; (**C**) 1,6-hexanedithiol; (**D**) 1,8-Octanedithiol.

**Figure 5:**
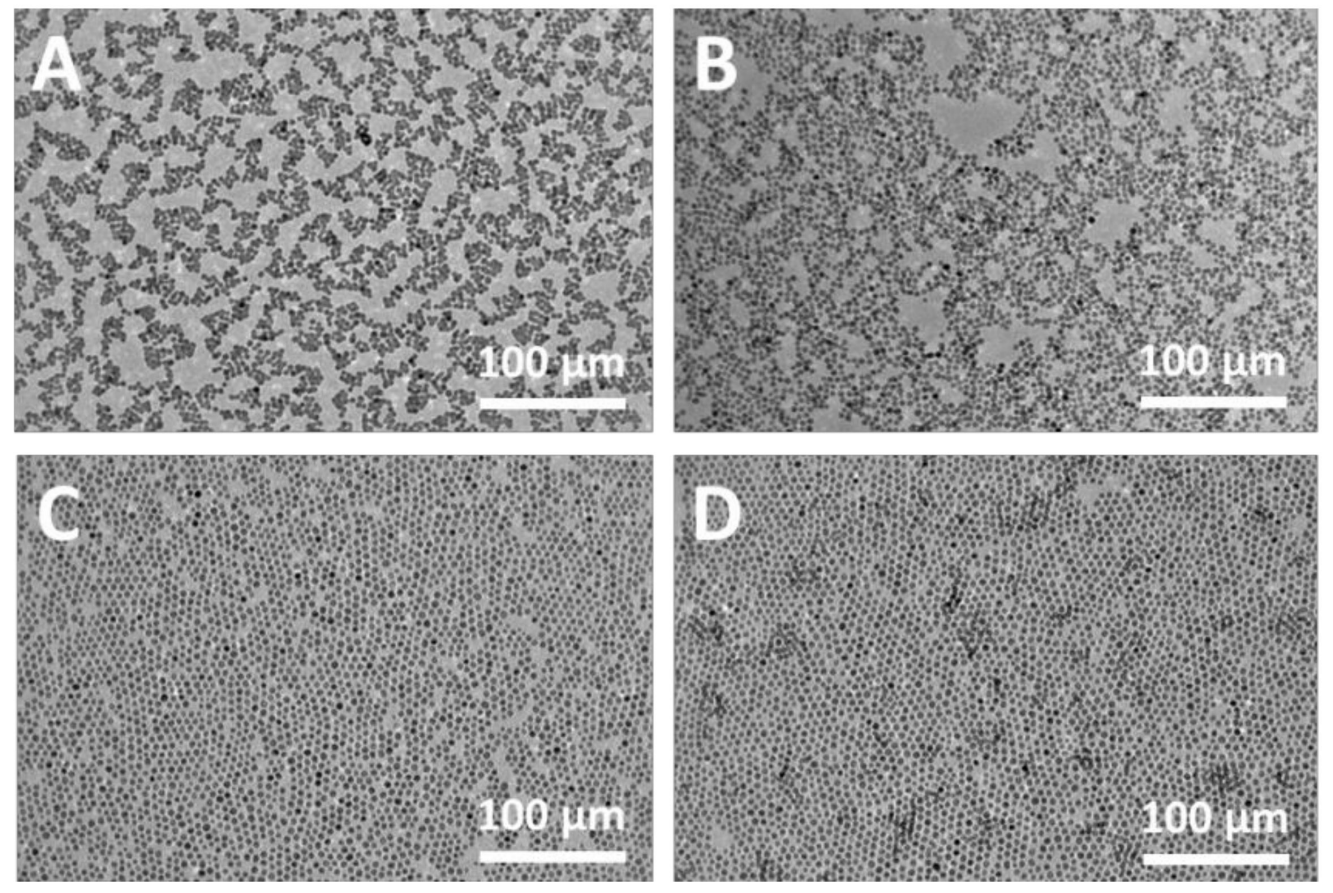
TEM images of the cross-linking monolayer array with different dithiol molecules on the Teflon substrate (**A**) 1,2-ethanedithiol; (**B**) 1,4-butanedithiol; (**C**) 1,6-hexanedithiol; (**D**) 1,8-octanedithiol.

To prove that this ligand exchange strategy has a general applicability, a similar cross-linking reaction based on the Au NP array was performed on the Teflon substrate. As shown in Figure 4, the color of the Au NP array gradually changed from bluish violet to green with a shorter dithiol, indicating a variation in the LSPR of the Au NP array. The TEM characterization results also suggested that the array became more compact after the cross-linking reaction, although some damaged holes formed in the array structure (deriving from the dithiol molecule stretching for nanoparticles), as shown in Figure 5. Therefore, the above study results clearly show that the ligand exchange strategy is not only suitable for different ligands, but is also appropriate for other substrates supporting the Au NP array.

**Figure 6:**
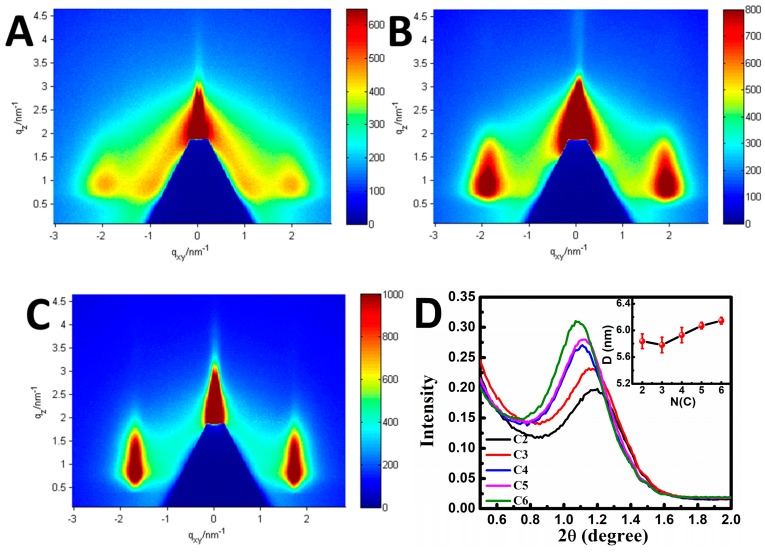
Two-dimensional GISAXS patterns obtained from an array cross-linked with (**A**) 1,2-ethanedithiol; (**B**) 1,4-butanedithiol; (**C**) 1,6-hexanedithiol. (**D**) Respective line cuts along the horizontal (qy) axis.

Apparently, the cross-linking array with a different internal structure could be obtained by this liquid exchanging system. In order to precisely appraise the variation of the array structure, a 2D GISAXS measurement was performed. As shown in Figure 6, there were only two diffraction arcs along the in-plane direction, which suggests that the Au NPs assembled to form a well-organized array parallel to the substrate. Comparing the three patterns, their diffraction arcs had a significant difference along the q-direction. The arcs became more blurred and shifted toward a larger q value with a shorter cross-linking dithiol, indicating the reduced ordering degree and interparticle spacing in the array. To quantitatively study the variation of interparticle spacing, 1D GISAXS was also employed to investigate the array structure. As shown in Figure 6D, the scattering peak value increased and the intensity weakened along with the decreased carbon numbers. The variation of the interparticle spacing against the carbon numbers of the dithiol is shown in the inset of Figure 6D. The interparticle distance gradually decreased from 6.14 nm to 5.83 nm after cross-linking with different dithiol molecules (C_2_–C_6_). These results sufficiently demonstrate that the interparticle spacing of the arrays could be regulated by variating the ligand length.

### 3.3. Mechanical Properties of the Cross-Linking Monolayer Film

**Figure 7:**
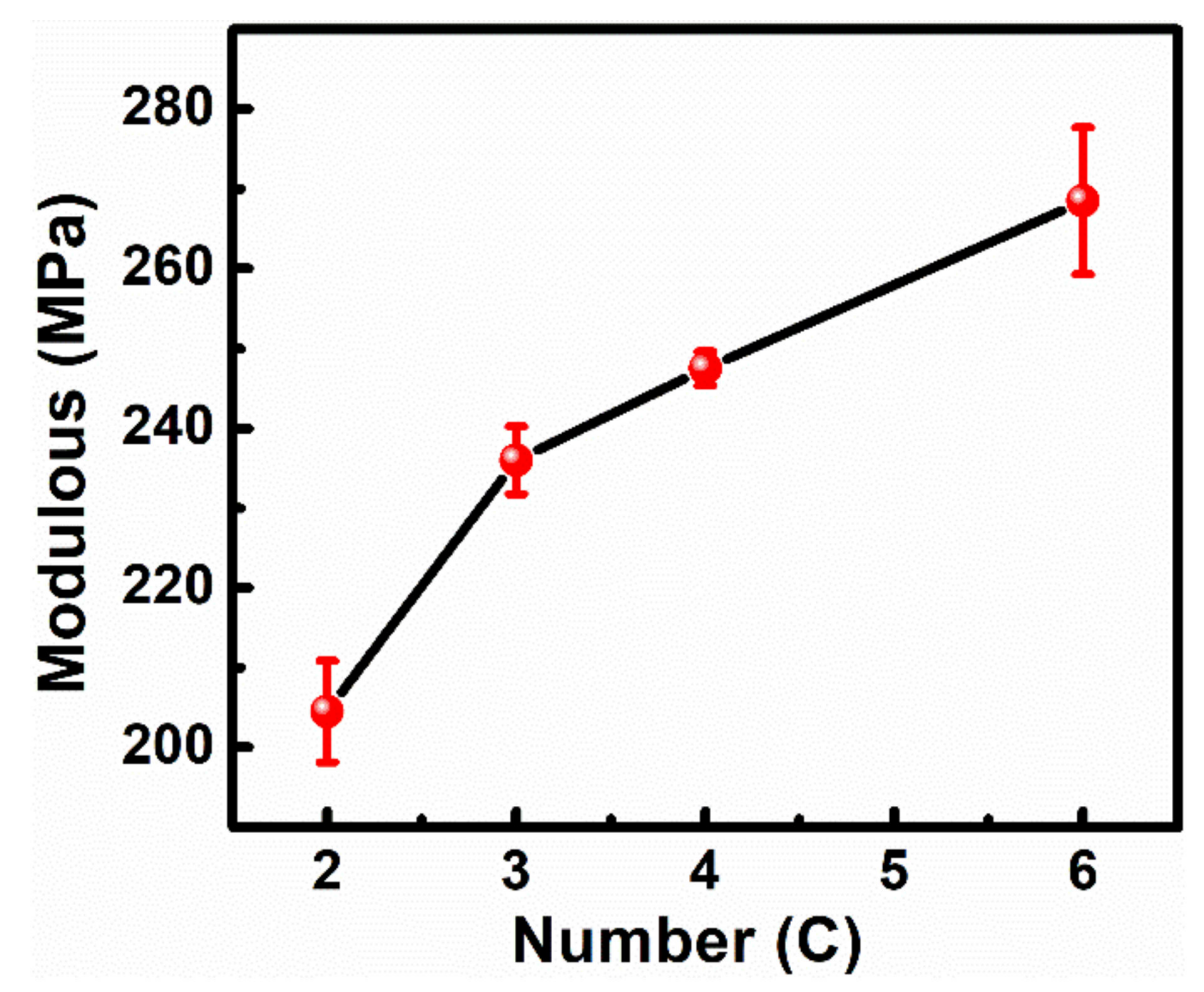
The Young’s modulus of the cross-linking monolayer film vs. the carbon numbers of the dithiol molecule.

As we all know, the mechanical properties of membranes are considerable for their application in various fields. For the self-assembled Au NP array, some non-covalent bonding forces (including van der Walls forces, electrostatic forces, etc.) dominate the main interactions between nanoparticles, which are relatively weak and easily influenced by the external surrounding. Here, the dithiol molecule linking Au NPs (Au–S bonding, the covalent bonding effect) could largely improve the interaction of adjacent nanoparticles. This generates a positive effect for the enhanced film strength. Although some studies have reported relevant work for strengthening the mechanical intensity of the membrane by the cross-linking reaction, systematic research on the modulus and cross-linking molecule has not been referred. Figure 7 shows the relationship between the ligand molecule length and Young’s modulus for the cross-linking array. Obviously, the membrane modulus improved significantly with the more carbon numbers of dithiol. The Young’s modulus of membrane (E_f_) is calculated according to the formula as follows:

E_f_ = 3E_s_(1 − V_f_^2^/1 − V_s_^2^) (d/2πh)^3^ (5)

where E_s_, V_f_, V_s_, d, and h represent the Young’s modulus of the PDMS, Poisson ratios of the membrane and PDMS, bucking wavelength, and membrane thickness, respectively. The Young’s modulus of membranes was calculated as 204.5 MPa, 236 MPa, 247.5 MPa, and 268.5 MPa, respectively, indicating an improvement in film mechanical property. We attribute this improved membrane strength to their microstructure. As discussed above, the cross-linking monolayer emerges with some broken holes in the array when using the short chain dithiol molecule as the cross-linking agent. This undesirable breakage generates a negative effect for the stability and strength of the membrane and finally presents the fragile property and weak strength. Therefore, the dithiol molecule with the appropriate carbon atom number is critical for its mechanical property. In our work, due to the approximate length between the dithiol chain length and interparticle spacing, the dithiol molecule with six carbon atoms cross-linking the nanoparticle array hardly changed the initial state of the array, which resulted in the integral structure was maintained as much as possible and the highest membrane strength (no damage in the structure). Here, the cross-linking technology is an available and useful strategy to strengthen the membranes’ intensity, and also offers an understanding of the membranes’ strengthening mechanism.

## 4. Conclusions

In summary, a cross-linking NP monolayer membrane was fabricated by the ligand exchange reaction. A series of dithiol molecules with different carbon numbers were used to replace the ligand molecule and successfully connected the adjacent nanoparticle, which led to the different microstructure of the nanoparticle monolayer. Additionally, this cross-linking manner exhibited a positive effect in enhancing the mechanical intensity of the membranes while having a general applicability for the other substrate. Moreover, we established a preliminary relationship between the ligands’ length and the membranes’ Young’s modulus, which offers a great reference in future applications as nano-devices.

## Figures and Tables

**Table 1:** Cross-linking ligands employed in the exchange reaction.

Types of Ligands	Concentrations (mmol·L^−1^)	Volume (μ L)
1,2-ethanedithiol (C_2_H_6_S_2_)	20	17
1,3-dimercaptopropane (C_3_H_8_S_2_)	20	20
1,4-Butanedithiol (C_4_H_10_S_2_)	20	23
1,5-pentanedithiol (C_5_H_12_S_2_)	20	27
1,6-hexanedithiol (C_6_H_14_S_2_)	20	30
